# Intranasal Vaccination With Lipoproteins Confers Protection Against Pneumococcal Colonisation

**DOI:** 10.3389/fimmu.2018.02405

**Published:** 2018-10-18

**Authors:** Franziska Voß, Thomas P. Kohler, Tanja Meyer, Mohammed R. Abdullah, Fred J. van Opzeeland, Malek Saleh, Stephan Michalik, Saskia van Selm, Frank Schmidt, Marien I. de Jonge, Sven Hammerschmidt

**Affiliations:** ^1^Department of Molecular Genetics and Infection Biology, Center for Functional Genomics of Microbes, Interfaculty Institute of Genetics and Functional Genomics, University of Greifswald, Greifswald, Germany; ^2^Department of Functional Genomics, Center for Functional Genomics of Microbes, Interfaculty Institute of Genetics and Functional Genomics, University Medicine Greifswald, Greifswald, Germany; ^3^Section Pediatric Infectious Diseases, Laboratory of Medical Immunology, Radboud Center for Infectious Diseases, Radboud Institute for Molecular Life Sciences, Radboud University Medical Center, Nijmegen, Netherlands; ^4^ZIK-FunGene, Department of Functional Genomics, Interfaculty Institute for Genetics and Functional Genomics, University Medicine Greifswald, Greifswald, Germany

**Keywords:** *Streptococcus pneumoniae*, lipoprotein, immunogenicity, colonization, protection

## Abstract

*Streptococcus pneumoniae* is endowed with a variety of surface-exposed proteins representing putative vaccine candidates. Lipoproteins are covalently anchored to the cell membrane and highly conserved among pneumococcal serotypes. Here, we evaluated these lipoproteins for their immunogenicity and protective potential against pneumococcal colonisation. A multiplex-based immunoproteomics approach revealed the immunogenicity of selected lipoproteins. High antibody titres were measured in sera from mice immunised with the lipoproteins MetQ, PnrA, PsaA, and DacB. An analysis of convalescent patient sera confirmed the immunogenicity of these lipoproteins. Examining the surface localisation and accessibility of the lipoproteins using flow cytometry indicated that PnrA and DacB were highly abundant on the surface of the bacteria. Mice were immunised intranasally with PnrA, DacB, and MetQ using cholera toxin subunit B (CTB) as an adjuvant, followed by an intranasal challenge with *S. pneumoniae* D39. PnrA protected the mice from pneumococcal colonisation. For the immunisation with DacB and MetQ, a trend in reducing the bacterial load could be observed, although this effect was not statistically significant. The reduction in bacterial colonisation was correlated with the increased production of antigen-specific IL-17A in the nasal cavity. Immunisation induced high systemic IgG levels with a predominance for the IgG1 isotype, except for DacB, where IgG levels were substantially lower compared to MetQ and PnrA. Our results indicate that lipoproteins are interesting targets for future vaccine strategies as they are highly conserved, abundant, and immunogenic.

## Introduction

*Streptococcus pneumoniae* continues to be a major cause of life-threatening invasive diseases such as pneumonia, sepsis and meningitis, especially in young children, the elderly and immunodeficient people (1). Two different types of vaccines are currently recommended by the World Health Organization (WHO) for the prevention of pneumococcal infections: the 23-valent polysaccharide vaccine (PPV23) and the pneumococcal conjugate vaccines PCV7, PCV10, and PCV13 (2). Despite their proven efficacy (3, 4), these vaccines have some important limitations, including restricted serotype coverage, which may facilitate replacement by non-vaccine serotypes, and high manufacturing costs (5–7). It is therefore vital to develop a new generation of vaccines, which can provide serotype-independent protection against pneumococcal infections, while being affordable for developing countries.

The pneumococcal cell-surface is decorated with a variety of proteins, which are exposed to the extracellular milieu of the host and are therefore the most promising targets for future protein-based vaccines. Consequently, pneumococcal surface proteins have been extensively studied over the last two decades, with the majority being characterised as virulence factors. Promising vaccine candidates, including PspA (Pneumococcal surface protein), PhtD (Pneumococcal histidine triad), PcpA (Pneumococcal choline-binding protein), PcsB (Pneumococcal cell wall separation protein), and StkP (serine/threonine protein kinase), have already been shown to be safe and immunogenic in clinical trials (8).

In this study, we particularly focussed on the lipoproteins, which are embedded in the pneumococcal cell membrane via a covalently anchored lipid moiety. Lipoproteins are highly conserved, and many of them influence pneumococcal fitness and virulence (9–14). Some studies have indicated the protective potential of lipoproteins against pneumococcal infections, with the well-characterised lipoprotein pneumococcal surface antigen A (PsaA), a manganese substrate-binding protein, being particularly in the research spotlight. PsaA is expressed by all serotypes of *S. pneumoniae* and is known to bind to human E-cadherin, thereby acting as an adhesin (15–19). Moreover, PsaA is highly immunogenic, as shown by the increased antibody responses that have been described as a result of pneumococcal exposure in children (20–22). Using intranasal challenge models in mice, PsaA has been shown to protect against pneumococcal carriage, demonstrated by reduced bacterial loads in the nasopharynx (23). A multivalent recombinant subunit protein vaccine containing PsaA, StkP, and PcsB was tested in a phase I trial (IC47, Intercell AG, Austria, NCT00873431) and shown to be safe and immunogenic (24, 25), resulting in the induction of protective antibodies against all three proteins. Besides PsaA, two other lipoproteins, SP_0148 and SP_2108, have emerged as promising vaccine candidates. Following intranasal immunisation, these proteins, which function as substrate-binding proteins for ABC transporters, showed protective efficacy in a mouse model of colonisation, which correlated with the observed elevation in IL-17A levels and depended on Toll-like receptor 2 signalling (26). Recently, Genocea Biosciences tested the GEN-004 vaccine (SP_0148, SP_2108 and SP_1912) using a human challenge model. Although the differences were not statistically significant, there was a trend in reducing carriage acquisition by 18–36% vs. the placebo (27), supporting the further development of GEN-004 and indicating the high potential of lipoproteins as components of a protein-based vaccine.

We therefore focused in our study on pneumococcal lipoproteins, aiming to identify new and promising candidates for a protein-based and serotype-independent vaccine. We analysed the immunogenicity of our candidates in mouse immunisation studies and by screening convalescent patient sera, while also assessing their abundance on the surface of pneumococci. It is essential that the antibodies raised by immunisation can recognise and bind to accessible surface proteins. Three lipoproteins were identified as the most promising candidates based on their high levels of conservation, their immunogenicity and their abundance on the pneumococcal cell-surface: the l,d-carboxypeptidase DacB (9), the methionine-binding protein MetQ (12), and the nucleoside-binding protein PnrA (11). DacB is a cell wall hydrolase and therefore essential for pneumococcal peptidoglycan turnover and the preservation of cell shape (9). MetQ and PnrA are substrate-binding lipoprotein components of the ABC transporters responsible for methionine or nucleoside uptake, respectively, from the extracellular space (11, 12). Pneumococcal mutants lacking these lipoproteins were previously shown to have significantly attenuated virulence in either systemic or pulmonary mouse infection models, although there is contradictory information regarding the role of MetQ in causing systemic infection in mice (9, 11, 12, 28, 29).

In our study, intranasal vaccination with these lipoproteins resulted in a reduced bacterial load in the nasal cavity, which correlated with increased nasal IL-17A levels. Humoral immune responses were characterised by high serum levels of IgG1 and substantial IgG2 levels, with the exception of the DacB vaccination. Our findings demonstrate the high potential of DacB and PnrA in particular for use in a future protein-based pneumococcal vaccine.

## Materials and methods

### Ethics statement

All animal experiments were conducted in accordance with the guidelines of the ethics committee at the University of Greifswald, the German regulations of the Society for Laboratory Animal Science (GV-SOLAS) and the European Health Law of the Federation of Laboratory Animal Science Associations (FELASA). All experiments were approved by the Landesamt für Landwirtschaft, Lebensmittelsicherheit und Fischerei Mecklenburg-Vorpommern (LALLF M-V, Rostock, Germany) and the LALLF M-V ethical board (LALLF M-V permit no. 7221.3-1-061/17). All efforts were made to minimise the discomfort of the animals and ensure the highest ethical standards.

### Bacterial strains, culture conditions, and pneumococcal mutant construction

*Streptococcus pneumoniae* wild-type and isogenic deletion mutants (Table 1) were grown on Columbia blood agar plates (Oxoid) supplemented with the appropriate antibiotics (50 μg/ml kanamycin, 5 μg/ml erythromycin or 10 μg/ml trimethoprim) and cultivated to mid-log phase (*A*_600_ = 0.35–0.40) in THY containing 36.4% Todd-Hewitt broth (Roth) and 0.5% yeast extract (Roth) at 37°C and in 5% CO_2_. *Escherichia coli* strains were cultured on solid Luria-Bertani (LB) medium plates or in liquid LB medium (Roth) to mid-log phase (*A*_600_ = 0.8) on an environmental shaker in the presence of kanamycin (50 μg/ml), ampicillin (100 μg/ml), and/or erythromycin (250 μg/ml) at 30°C. To generate the Δ*metQ (sp_0149)*, Δ*sp_0191*, Δ*pnrA (sp_0845)*, Δ*sp_0899*, and Δ*adcAII (sp_1002)* mutants, the loci of the respective genes and their upstream and downstream flanking sequences were amplified from *S. pneumoniae* TIGR4 genomic DNA using PCR and the primer pairs listed in Table 2. Following the manufacturer's instructions, the PCR products were directly cloned into pGEM®-T Easy vectors (Promega, Madison, WI, USA) and transformed into *E. coli* DH5α competent cells. The recombinant plasmids p559, p560, p576, p573, and p572 harbouring the desired DNA inserts were purified and used as templates for inverse PCR reactions with the primer pairs listed in Table 2. The deleted sequences were replaced with the *ermB* resistance gene, which was amplified from plasmid pE89 using PCR with the primer pair ermB_105/ermB_106 (Table 2). These recombinant plasmids were used to transform pneumococci, as described previously (35). The non-encapsulated pneumococcal mutants D39Δ*psaA*, D39Δ*pspA*, D39Δ*ppmA*, and D39Δ*slrA* were generated by transformation with the recombinant plasmid p873, in which the capsule gene locus is replaced by the *aphA3* resistance gene.

**Table 1:** Strain and plasmid list.

**Strain/plasmid**	**Serotype and relevant genotype**	Resistance^*^	**Source or reference**
***Streptococcus pneumoniae***			
SP257 (D39)	2	None	NCTC7466
PN111	D39Δ*cps*	Km^r^	(30, 31)
PN282	D39Δ*cps*Δ*adcAII* (*spd_0888*)	Km^r^, Erm^r^	This work
PN279	D39Δ*cps*Δ*dacB* (*spd_0549*)	Km^r^, Erm^r^	(9)
PN253	D39Δ*cps*Δ*etrx1* (*spd_0572*)	Km^r^, Erm^r^	(10)
PN281	D39Δ*cps*Δ*etrx2* (*spd_0886*)	Km^r^, Erm^r^	(10)
PN238	D39Δ*cps*Δ*metQ* (*spd_0151*)	Km^r^, Erm^r^	This work
PN732	D39Δ*cps*Δ*ppmA* (*spd_0868*)	Km^r^, Trm^r^	This work
PN280	D39Δ*cps*Δ*pnrA* (*spd_0739*)	Km^r^, Erm^r^	This work
PN301	D39Δ*cps*Δ*psaA* (*spd_1463*)	Km^r^, Erm^r^	This work
PN733	D39Δ*cps*Δ*slrA* (*spd_0672*)	Km^r^, Erm^r^	This work
PN241	D39Δ*cps*Δ*spd_0179*	Km^r^, Erm^r^	This work
PN312	D39Δ*cps*Δ*spd_0792*	Km^r^, Erm^r^	This work
PN735	D39Δ*cps*Δ*pspA* (*spd_0126*)	Km^r^, Erm^r^	This work
PN278	D39Δ*adcAII* (*spd_0888*)	Erm^r^	This work
PN275	D39Δ*dacB* (*spd_0549*)	Erm^r^	(9)
PN246	D39Δ*etrx1* (*spd_0572*)	Erm^r^	(10)
PN277	D39Δ*etrx2* (*spd_0886*)	Erm^r^	(10)
PN311	D39Δ*metQ* (*spd_0151*)	Erm^r^	This work
PN093	D39Δ*ppmA* (*spd_0868*)	Trm^r^	(32)
PN276	D39Δ*pnrA* (*spd_0739*)	Erm^r^	This work
PN172	D39Δ*psaA* (*spd_1463*)	Erm^r^	(13)
PN095	D39Δ*slrA* (*spd_0672*)	Erm^r^	(32)
PN243	D39Δ*spd_0179*	Erm^r^	This work
PN251	D39Δ*spd_0792*	Erm^r^	This work
PN031	D39Δ*pspA* (*spd_0126*)	Erm^r^	(33)
***Escherichia coli***			
DH5α	Δ*(lac)U169, endA1, gyrA46, hsdR17, Φ80*Δ*(lacZ)M15, recA1, relA1, supE44, thi-1*	None	Bethesda Research Labs, Gaithersburg, U.S.
BL21(DE3)	*E. coli* B, *F- dcm ompT hsdS gal λ*(DE3), T7 polymerase gene under control of the lacUV5 promoter	None	Novagen, Merck KGaA, Darmstadt, Germany
**Plasmids**			
pGEM®-T easy	TA cloning vector for PCR products	Ap^r^	Madison, U.S.
p89	pCR2.1Topo with erythromycin (*ermB*) cassette	Ap^r^, Km^r^, Erm^r^	(34)
p873	pGXT with capsule locus replaced by *aphA3* gene resistance cassette, flanking genes *dex* ,and *aliA*	Ap^r^, Km^r^	(10)
p572	pGEM-T derivative with *sp_1002* (*adcAII*) 5′ and 3′ flanking region for mutagenesis	Ap^r^	This work
p598	pGEM-T derivative with *sp_1002* (*adcAII*) interrupted by *ermB* gene resistance cassette	Ap^r^, Erm^r^	This work
p559	pGEM-T derivative with *sp_0149* (*metQ*) 5′ and 3′ flanking region for mutagenesis	Ap^r^	(28)
p563	pGEM-T derivative with *sp_0149* (*metQ*) interrupted by *ermB* gene resistance cassette	Ap^r^, Erm^r^	(28)
p576	pGEM-T derivative with *sp_0845* (*pnrA*) 5′ and 3′ flanking region for mutagenesis	Ap^r^	This work
p646	pGEM-T derivative with *sp_0845* (*pnrA*) interrupted by *ermB* gene resistance cassette	Ap^r^, Erm^r^	This work
p560	pGEM-T derivative with *sp_0191* (*spd_0179*) 5′ and 3′ flanking region for mutagenesis	Ap^r^	This work
p565	pGEM-T derivative with *sp_0191* (*spd_0179*) interrupted by *ermB* gene resistance cassette	Ap^r^, Erm^r^	This work
p573	pGEM-T derivative with *sp_0899* (*spd_0792*) 5′ and 3′ flanking region for mutagenesis	Ap^r^	This work
p577	pGEM-T derivative with *sp_0899* (*spd_0792*) interrupted by *ermB* gene resistance cassette	Ap^r^, Erm^r^	This work
pTP1	pET28 expression vector, N-terminal His-tag, TEV protease cleavage site, induction by IPTG	Km^r^, Erm^r^	(10)
p648	pTP1 with TIGR4 *sp_1002* (*adcAII*) for protein production and mice immunisation	Km^r^	This work
p652	pTP1 with TIGR4 *sp_0629* (*dacB*) for protein production and mice immunisation	Km^r^	(9)
p629	pTP1 with TIGR4 *sp_0659* (*etrx1*) for protein production and mice immunisation	Km^r^	(10)
p651	pTP1 with TIGR4 *sp_1000* (*etrx2*) for protein production and mice immunisation	Km^r^	(10)
P732	pTP1 with TIGR4 *sp_0149* (*metQ*) for protein production and mice immunisation	Km^r^	This work
p264	pET11a with R6 *spr0884* (*ppmA*) for protein production and mice immunisation	Km^r^	(32)
p649	pTP1 with TIGR4 *sp_0845* (*pnrA*) for protein production and mice immunisation	Km^r^	This work
p653	pTP1 with TIGR4 *sp_1650* (*psaA*) for protein production and mice immunisation	Km^r^	This work
p263	pET11a with R6 *spr0679* (*slrA*) for protein production and mice immunisation	Km^r^	(32)
p628	pTP1 with TIGR4 *sp_0191* (*spd_0179*) for protein production and mice immunisation	Km^r^	This work
p631	pTP1 with TIGR4 *sp_0899* (*spd_0792*) for protein production and mice immunisation	Km^r^	This work
p105	pQE30 with PspA (aa 32-289) without choline-binding domain	Ap^r^	(33, 68)

* *Km^r^, Kanamycin; Erm^r^, Erythromycin; Ap^r^, Ampicillin; Trm^r^, Trimethoprim*.

**Table 2:** Primer list.

**Primer use**	**Primer**	Sequence (5′-3′)^*^
**INSERTION-DELETION MUTAGENESIS**		
Amplification of *sp_1002* + 5′ and 3′ flanking region	adcAII_427 adcAII_430	5′-CTACTA GAATTCGATGATGCCGTTGCCTTT-3′ 5′-TTCCAAGCTGCAGATCCCTGCTTCCCATTCC-3′
Inverse PCR of *sp_1002* + 5′ and 3′ flanking region (pGEM-T Easy)	adcAII_429 adcAII_428	5′-CTCACTGAAGCTTGACCCACAAAATGACAAGACC-3′ 5′-ATCATCGAAGCTTCCCCCAAGCACAAAAGAA-3′
Amplification of *sp_0149* + 5′ and 3′ flanking region	metQ_382 metQ_385	5′-CTACTACTAGAATTCATGCTGAACACACGGACAAC-3′ 5′-AACCTTCCAAGCTGCAGCCGCTCCCTCCATGATAAAG-3′
Inverse PCR of *sp_0149* + 5′ and 3′ flanking region (pGEM-T Easy)	metQ_384 metQ_383	5′-ACTCACTCACTGAAGCTTATCGCAGCTTACCACACAGA-3′ 5′-ATCATCATCATCGAAGCTTAGCCAAACCTGCGACTGTAG-3′
Amplification of *sp_0845* + 5′ and 3′ flanking region	pnrA_415 pnrA_418	5′-CTACTAGAATTCAAAAAGCTGGGGCTGAC-3′ 5′-CCAAGCTGCAGCGGTCAGAAACTGCTCGAAT-3′
Inverse PCR of *sp_0845* + 5′ and 3′ flanking region (pGEM-T Easy)	pnrA_417 pnrA_416	5′-CTCACTGCTCGAGTGGAAGCGTAAAAGTTCCTGA-3′ 5′-ATCATCGGTACCGAGCGGTTACCACATGCAG-3′
Amplification of *sp_0191* + 5′ and 3′ flanking region	sp_0191_392 sp_0191_395	5′-CTACTACTAGAATTCATGTAGCGAAAGGGGTAGG-3′ 5′-AACCTTCCAAGCTGCAGCTTTGCTCCGTAGGCTTGAC-3′
Inverse PCR of *sp_0191* + 5′ and 3′ flanking region (pGEM-T Easy)	sp_0191_394 sp_0191_393	5′-ACTCACTCACTGAAGCTTATGGCGCGACAGAACAATAG-3′ 5′-ATCATCATCATCGAAGCTTAACCAACCAGGACAAAAAGG-3′
Amplification of *sp_0899* + 5′ and 3′ flanking region	sp_0899_419 sp_0899_422	5′-CTACTAGAATTCCCTTGTCTGGGTGGTTCC-3′ 5′-CCAAGCTGCAGTGGGACTAGCGCCAGAA-3′
Inverse PCR of *sp_0899* + 5′ and 3′ flanking region (pGEM-T Easy)	sp_0899_421 sp_0899_420	5′-CTCACTG*CTCGAG*GCGAGGGACTGGCTAA-3′ 5′-ATCATCGGTACCCAAGCAGCCAAGCCTAAAA-3′
**ANTIBIOTIC CASSETTE AMPLIFICATION**		
Erythromycin (*ermB*)	ermB_105	5′-GATGATGATGATCCCGGGTACCAAGCTTGAATTCACGGTTCGTGTTCGTGCTG-3′
	ermB_106	5′-AGTGAGTGAGTCCCGGGCTCGAGAAGCTTGAATTCGTAGGCGCTAGGGACCTC-3′
**RECOMBINANT PROTEIN PRODUCTION**		
*sp_0899* (TIGR4)	sp_0899_463 sp_0899_464	5′-GCGCGCTAGCCAACAACAACATGCTACTTC-3′ 5′-GGCCGAGCTCTTAAAGTTTAACCCACTTATC-3′
*sp_1002* (TIGR4; *adcAII*)	adcAII_451 adcAII_452	5′-GGGCGCTAGCGGTCAAAAGGAAAGTCAGAC-3′ 5′-GCGGCCAAGCTTACTTTAATTCTTCTGCTAG-3′
*sp_0149* (TIGR4; *metQ*)	metQ_410 metQ_391	5′-AAAGCATATGAGCGGCGAAAACCTGTATTTTCAGGGCGCTAGCGGAAACTCAGAAAAGAAAGC-3′ 5′-CCAACCTTCCAAGCTTACCAAACTGGTTGATCC-3′
*sp_0845* (TIGR4; *pnrA*)	pnrA_449 pnrA_450	5′-AAGCGCTAGCGGTAACCGCTCTTCTCGTA-3′ 5′-GGGGCCAAGCTTATTTTTCAGGAACTTTTACGC-3′
*sp_1650* (TIGR4; *psaA*)	psaA_488 psaA_489	5′-GCGCGCTAGCGGAAAAAAAGATAC-3′ 5′-GCGCAAGCTTATTTTGCCAATCCTTCAG-3′
*sp_0191* (TIGR4)	sp_0191_392 sp_0191_393	5′-TATTTTCAGGGCGCTAGCGGACAGAAAAAAGAAACTGG-3′ 5′-CCAACCTTCCAAGCTTATTGTTCTGTCGCGCCATTTG-3′

* *Restriction sites used for cloning are underlined*.

### Heterologous expression, purification of recombinant proteins, and production of polyclonal antisera

**Figure 1:**
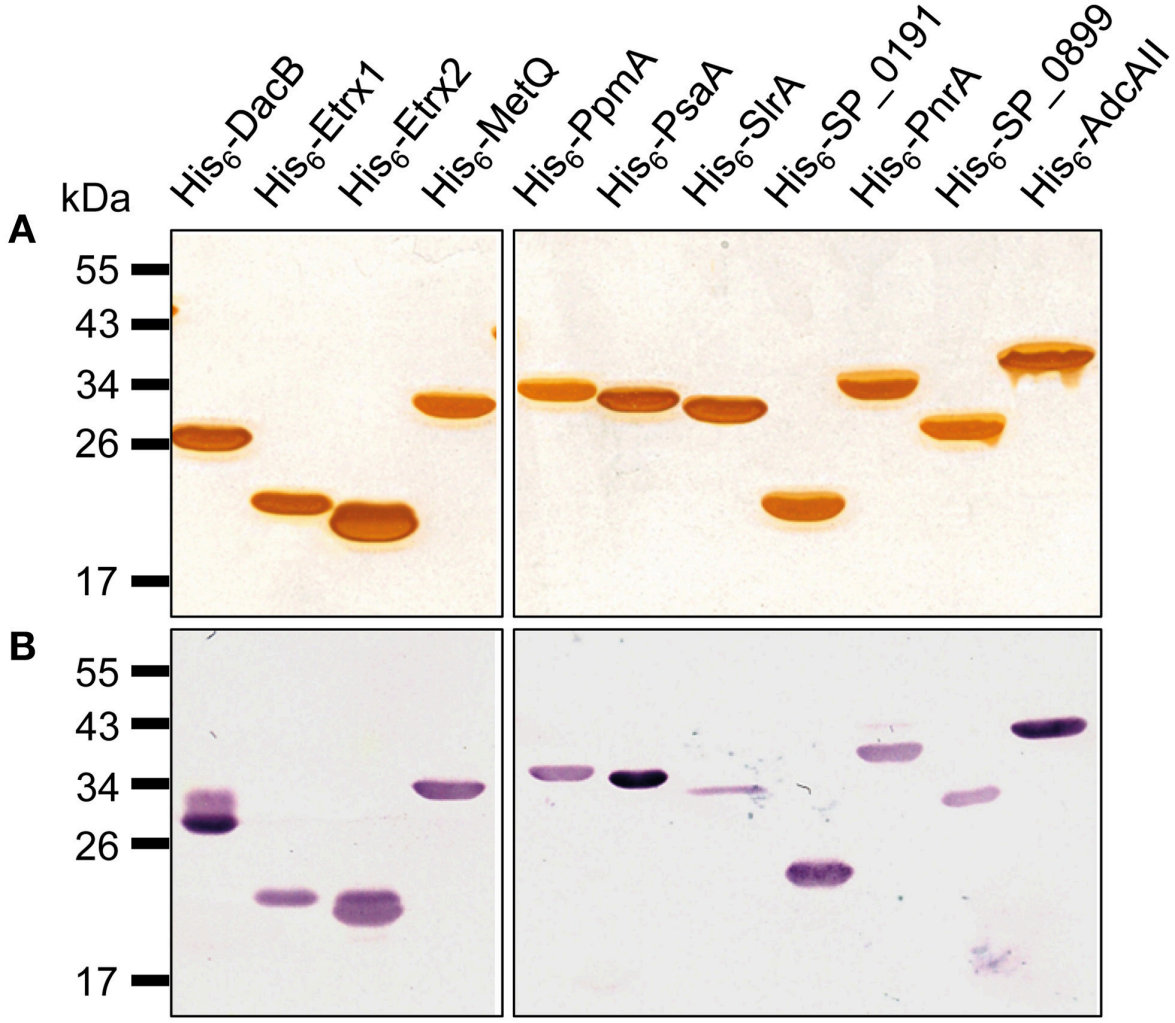
Pneumococcal lipoproteins heterologously expressed *in E. coli*. A 1-μg aliquot of heterologously expressed pneumococcal lipoproteins was separated using SDS-PAGE and the proteins were detected using silver staining **(A)** or with immunoblotting using a monoclonal mouse anti-Penta-His_6_ antibody and alkaline phosphatase-conjugated goat anti-mouse IgG **(B)**.

The N-terminally His_6_-tagged proteins used in this study were either described previously (Table 2) or were generated by cloning the PCR products of the target genes *metQ, sp_0191, pnrA, sp_0899*, and *adcAII*, without their signal sequences, into the pTP1 expression vector (10). PCR reactions were performed using *S. pneumoniae* TIGR4 chromosomal DNA as a template and the primer pairs listed in Table 2. The primers contained restriction sites (*Nhe*I/*Sac*I, *Nhe*I/*Hin*dIII, or *Nde*I/*Hin*dIII), which were used to ligate the fragments into similarly digested expression vectors. The resulting plasmids (Table 1) were transformed into competent *E. coli* BL21 (DE3). For protein production, the recombinant *E. coli* BL21 (DE3) were cultured in LB, supplemented with kanamycin (50 μg/ml) or ampicillin (100 μg/ml), to an *A*_600_ of 0.6–0.8 at 30°C. Protein expression was induced with 1 mM IPTG (isopropyl-β-D-1-thiogalactopyranoside; Hartenstein, Wuerzburg, Germany) and the cells were cultured for another 3 h. The resulting His_6_-tagged proteins were purified using affinity chromatography in a His Trap™ HP Ni-NTA column (1 ml; GE Healthcare, Chicago, IL, USA) on the ÄKTA Purifier liquid chromatography system (GE Healthcare), following the manufacturer's instructions. Purified proteins were dialysed (12–14 kDa molecular weight cut off) against phosphate-buffered saline (PBS; pH 7.4). The absorbance of the proteins was determined at *A*_280_ using a NanoDrop® ND-1000 (Thermo Fisher Scientific, Waltham, MA, USA) to calculate the protein concentrations while considering the extinction coefficients and molecular weights. After sodium dodecyl sulphate polyacrylamide gel electrophoresis (SDS-PAGE), the purity of the proteins was analysed using silver staining and immunoblotting (Figure 1) with an anti-Penta-His-tag mouse antibody (Qiagen, Hilden, Germany). The recombinant proteins were used in intraperitoneal immunisations using Imject™ Alum as an adjuvant (Thermo Fisher Scientific). Six- to eight-week-old female CD-1 mice (Charles River Laboratories, Sulzfeld, Germany) were vaccinated by intraperitoneal injection with 100 μl of a 1:1 emulsion containing 20 μg recombinant protein and the adjuvant. The mice received vaccine boosters at days 14 and 28 and were bled after 6 weeks. Serum samples were taken before each immunisation step (pre-immune, priming, 1st boost) and 2 weeks after the third immunisation (post-immune) and stored at −20°C until use.

### Purification of polyclonal IgG and immunoblotting

Polyclonal IgGs were purified from the generated antisera using protein A-sepharose chromatography. Protein A sepharose CL-4B columns (GE Healthcare), stored in 20% ethanol at 4°C, were equilibrated in binding buffer (50 mM Tris-HCl, pH 7.0). After mixing the antisera with one volume of binding buffer, the mixture was applied to the column and incubated for 15 min at room temperature (RT). The column was washed with binding buffer until the absorption measured using a NanoDrop® ND-1000 dropped below *A*_280_ = 0.05. The elution was performed using 1-ml aliquots of elution buffer (100 mM glycine, pH 3.0) collected in 50 μl phosphate buffer (50 mM K_2_HPO_4_, pH 8.5). The IgG concentration of each sample was determined by measuring the absorption at *A*_280_ using a NanoDrop® ND-1000. Immunoblots were carried out to analyse the specificity of the purified polyclonal IgGs. After the SDS-PAGE-mediated separation of bacterial lysates from late exponential growth phase cells, the proteins were transferred onto a nitrocellulose membrane using semidry blotting (Bio-Rad Laboratories, Hercules, CA, USA). The membrane was blocked with 5% skim milk (in Tris-buffered saline (TBS), Roth) overnight at 4°C. Following an incubation with mouse polyclonal IgGs (1:1,000 in blocking buffer) recognising lipoproteins or rabbit anti-enolase serum (1:25,000 in blocking buffer) for 1 h at RT, the membrane was washed three times with washing buffer (TBS, 0.05% Tween® 20). A secondary antibody, goat anti-mouse IgG (Dianova, Hamburg, Germany; 1:5,000) or goat anti-rabbit IgG (Dianova; 1:5,000) horseradish peroxidase conjugate was used for 1 h at RT, then washed three times with washing buffer. Finally, antibody binding was detected using an enhanced chemiluminescence reaction (luminol and p-coumaric acid, Roth).

### Antibody titration of polyclonal IgGs using enzyme-linked immunosorbent assays (ELISAs) and the flow cytometric analysis of surface abundance

The antibody titres of polyclonal IgGs were determined using ELISAs. Microtiter plates (96-well, PolySorp®, Nunc, Thermo Fisher Scientific) were coated with equimolar amounts of pneumococcal proteins (30 pmol/well) overnight at 4°C. The plates were washed three times with washing buffer (PBS, pH 7.4, 0.05% Tween® 20) and blocked with blocking buffer (PBS, 0.1% Tween® 20 supplemented with 2% bovine serum albumin) for 1 h at RT. The wells were washed and incubated for 1 h at RT with polyclonal IgGs in serial dilutions ranging from 1:750 to 1:24,000 in blocking buffer. Antibody binding was detected using goat anti-mouse IgG coupled to horseradish peroxidase (1:1,000, Jackson ImmunoResearch Laboratories, Inc., Ely, UK) as the secondary antibody (1 h incubation at RT). For the detection, 0.03% H_2_O_2_ and *o*-phenylenediamine dihydrochloride (OPD, Agilent Technologies, Santa Clara, CA, USA) at a final concentration of 0.67 mg/ml were used in a colorimetric reaction, which was stopped by adding 2M H_2_SO_4_. The absorbance of each sample was measured at *A*_492_ using the FLUOstar Omega Microplate Reader (BMG Labtech, Ortenberg, Germany). This resulted in hyperbolic titration curves ($y=\frac{Bmax\cdot x}{Kd+x}$, *Bmax*, maximal binding; *Kd*, concentration for half the maximal binding), which were used to calculate the relative IgG concentrations. The absorbance was therefore set to *A*_492_ = 0.3 (y) in the linear dynamic range and the IgG concentrations (x) were calculated and denoted as the 1× end concentration in the flow cytometry. The polyclonal IgGs with equal contents of IgGs specific to the lipoproteins were therefore applied to enable the comparison of surface abundances.

For flow cytometry, *S. pneumoniae* wild-type D39, its capsule-deficient derivative (D39Δ*cps*) and the isogenic mutants were cultured in 30 ml THY to *A*_600_ 0.35–0.4. The bacteria were washed with PBS, then resuspended in 1 ml of PBS. To detect the proteins on the surface of the pneumococci, 2 × 10^8^ bacteria were incubated with mouse polyclonal IgG (1×, 5×, 10×, 20×, and 50× end concentration in PBS) for 45 min at 4°C. The bacteria were washed with PBS and stained using secondary antibody goat anti-mouse IgG Alexa-Fluor-488 conjugate (1:1,000; Thermo Fisher Scientific). After another 45-min incubation at 4°C, the bacteria were washed with PBS and fixed with 1% paraformaldehyde overnight at 4°C. The flow cytometry was conducted using a FACSCalibur™ (BD Biosciences, Heidelberg, Germany), and the CellQuestPro Software 6.0 (BD Biosciences) was used for data acquisition. The data were analysed using Flowing Software 2.5.1 (by Perttu Terho, Turku Centre for Biotechnology). The bacteria were detected and gated as described previously (36).

### Intranasal immunisation and pneumococcal challenge of mice

Seven-week-old female C57BL/6 mice (*n* = 12; Charles River Laboratories) were intranasally immunised three times at 2-week intervals under anaesthesia (50 mg ketamine and 5 mg xylazine per kg mouse weight). The 10 μl vaccine contained 5 μg recombinant DacB, MetQ, PnrA, or PspA proteins in combination with 4 μg cholera toxin subunit B (CTB; Sigma-Aldrich, St. Louis, MO, USA) in PBS. Control mice were mock-treated with an equivalent volume of PBS and adjuvant. Three weeks after the last vaccination, the mice were infected with 10 μl PBS containing 3.4 × 10^6^ CFU of *S. pneumoniae* D39. Three days after the bacterial challenge, the mice were euthanised and their blood and nasal tissues were harvested. Nasal tissue was homogenised in 1 ml PBS using a T10 basic blender (IKA, Staufen, Germany), and serially diluted samples were plated on blood agar (Oxoid) to quantify the recovered bacteria (log CFU/ml). Serum samples were taken before each immunisation step (pre-immune, priming, 1st boost), 2 weeks after the third immunisation (post-immune) and after the challenge with pneumococci. They were stored at −20°C until use.

### Detection of local IL-17A in the nasopharyngeal-associated lymphoid tissue (NALT)

Cytokine production in mouse nasal samples was determined using a bead-based immunoassay (Bio-Rad Laboratories), according to manufacturer's instructions. The assay was performed using the Bio-Plex Pro™ Reagent Kit, the Bio-Plex Pro™ mouse cytokine IL-17A set and the Bio-Plex Pro™ mouse cytokine standard group I, 23-Plex. The concentrations were calculated using Graph Pad Prism 5.

### Local and systemic antibody and isotype levels

Local IgG and IgA levels in the nasal tissue and the systemic total levels of IgG, IgG1, and IgG2a/IgG2c isotype were determined in the post-immune (Alum immunisation) or post-challenge sera (CTB immunisation) using an ELISA. PolySorp® Microtiter plates (96-well, Nunc, Thermo Fisher Scientific) were coated with equimolar amounts of pneumococcal proteins (3 pmol/well) and stored overnight at 4°C. The plates were washed and blocked as described above, then the wells were incubated with samples diluted in blocking buffer for 1 h at 37°C. The IgA and IgG levels in the nasal tissue samples were detected using 1:2 and 1:10 dilutions, respectively. Post-immune (immunisation only) and post-challenge sera (immunisation and challenge) were serially diluted ranging from 1:100 to 1:60,000 or 1:50 to 1:10,000, respectively. The plates were washed and incubated for 1 h at RT with horseradish peroxidase coupled with rabbit anti-mouse total IgG (Jackson ImmunoResearch Laboratories, Inc.), rabbit anti-mouse IgG1 (Sigma-Aldrich), goat anti-mouse IgG2a (Sigma-Aldrich), goat anti-mouse IgG2c (Abcam, Cambridge, UK), or goat anti-mouse IgA antibody (Sigma-Aldrich). The protection study using CTB as an adjuvant was carried out in C57BL/6 mice, which express IgG2c instead of IgG2a due to a gene replacement (37). These mice were therefore isotyped for IgG2c rather than the IgG2a used for the CD-1 mice. Detection was performed as described above. The antibody titre of each serum specimen was denoted as the log_10_ of its reciprocal dilution of the serum giving twice the average absorbance of the sera derived from the PBS-treated group.

### Monitoring of antibody titres directed against pneumococcal lipoproteins using a FLEXMAP 3D® analysis

The bead-based flow cytometric technique FLEXMAP 3D® (Luminex Corporation) was applied to simultaneously quantify the antibodies directed against the 12 pneumococcal surface proteins. This analysis was carried out as described recently (38), using the same commercially available reagents and instruments. Purified His_6_-tagged proteins were covalently coupled to 6.25 × 10^5^ fluorescent FLEXMAP 3D® MagPlex® beads. The beads were protected from light throughout the workflow to avoid photo bleaching, and all incubation steps were carried out under agitation (900 rpm). After three washes with 100 mM monobasic sodium phosphate (activation buffer, pH 6.2) using a magnetic 96-well separator, the carboxyl groups on the surface of the beads were activated for 20 min by a resuspension in activation buffer (5 mg/ml each of EDC and sulpho-NHS). The activated beads were washed three times with coupling buffer [50 mmol/l 2-(*N*-morpholino)ethanesulphonic acid, pH 5.0] followed by a 2-h incubation with 125 μl recombinant *S. pneumoniae* protein solution (100 μg/ml). The coupled beads were washed three times with washing buffer (PBS, 0.05% (v/v) Tween® 20, pH 7.4) and adjusted to a concentration of 125 beads per μl using blocking-storage buffer (1% (w/v) bovine serum albumin and 0.05% (v/v) ProClin™ 300 in PBS, pH 7.4). The beads were stored at 4°C until use. A coupling control was applied to validate the coupling efficiency. A master mix of sonicated coupled beads was prepared by diluting the beads 1:50 in bead buffer [50% (v/v) blocking-storage buffer and 50% (v/v) LowCross-Buffer® (LCB)]. After incubation with the anti-Penta-His tag mouse antibody (final concentration 10 μg/ml) for 45 min, the beads were washed three times with washing buffer and stained for 30 min using R-phycoerythrin (RPE)-conjugated goat anti-mouse IgG (final concentration 5 μg/ml). After another washing procedure, the beads were resuspended in 100 μl xMAP® Sheath Fluid and measured in the Luminex^®^ FLEXMAP 3D® system with the following instrumental setup: sample size 80 μl, sample timeout 60 s, bead count 10,000, and gate settings 7,500–15,000 under standard PMT (Photomultiplier tube) settings.

A multiplex immunoassay was used to compare differences in the amounts of anti-pneumococcal antibodies in serum samples obtained from 22 patients convalescent from pneumococcal infections. Convalescent-phase sera were kindly provided by Gregor Zysk, University of Düsseldorf, Germany (39). The infections included pneumonia (*n* = 6), meningitis (*n* = 7), sepsis (*n* = 4), and unknown clinical outcomes (*n* = 5), which were all caused by different pneumococcal serotypes. In addition, post-immune sera (2 weeks after the third immunisation) were obtained from CD-1 mice (*n* = 6) intraperitoneally immunised with Imject™ Alum or C57BL/6 mice intranasally immunised with CTB as an adjuvant, and were analysed to determine their antibody titres for the indicated proteins.

For the multiplex assay, serum samples were serially diluted (1:50, 1:500, 1:1,000, 1:10,000, 1:25,000, 1:50,000 and 1:100,000) in assay buffer (90% (v/v) bead buffer and 10% (v/v) *E. coli* BL21 lysate) and incubated for 20 min at RT to block unspecific binding. A bead master mix was prepared with a bead count of 1,000 per well. A 50-μl aliquot of the diluted sample was added to the beads and incubated overnight at 4°C, after which the beads were washed three times with 100 μl washing buffer. The beads were incubated with 50 μl RPE-conjugated goat anti-human IgG (final concentration 5 μg/ml) or 50 μl RPE-conjugated goat anti-mouse IgG (final concentration 5 μg/ml) for 1 h at RT. After three washes with 100 μl washing buffer, the beads were resuspended in 100 μl xMAP® Sheath Fluid (Invitrogen™) and measured in the Luminex® FLEXMAP 3D® system using the following instrumental setup: sample size 80 μl, sample timeout 60 s, bead count 100, and gate settings 7,500–15,000 under standard PMT.

The data were analysed as described recently (38). Following Clark's theory interaction model, the titration curves were used to determine the percentage of the half-maximal MFI (mean fluorescence intensity) for the respective coupling control. The MFI was multiplied with the reciprocal serum dilution corresponding to the half-maximal MFI. The resulting MFI values reflect the antigen binding intensity of antibodies contained in each serum sample. Calculations were performed using R (package 3.0.1) or GraphPad Prism version 5.0 (GraphPad Software).

### Statistical analysis

All statistical analyses were performed using GraphPad Prism version 5.0 (GraphPad Software). The one-way ANOVA Kruskal-Wallis test with Dunn's post-test was used to compare multiple groups, while a Mann-Whitney *U*-test was used to compare two groups in the analysis of the protection efficacy and IL-17A levels in the nasal tissues or local/systemic humoral immune responses.

## Results

### Selection and purification of pneumococcal lipoproteins

A previous *in silico* analysis of the pneumococcal genome (*S. pneumoniae* strain D39) predicted more than 100 surface-associated or secreted proteins, including 37 lipoproteins (40). We selected 11 of these lipoproteins based on the following criteria: (i) confirmed member of the lipoprotein cluster (40), (ii) expressed in pneumococci (11, 17, 40), and (iii) high levels of conservation (>85%, Table 3). The selected lipoproteins included the four substrate-binding proteins AdcAII, MetQ, PnrA, and PsaA, which are components of ABC transporters responsible for the uptake of zinc(II), methionine, nucleosides and manganese(II), respectively. We also selected the following non-ABC transporter lipoproteins: (i) the thioredoxins Etrx1 and Etrx2, which are involved in pneumococcal resistance to oxidative stress, (ii) the l,d-carboxypeptidase DacB, (iii) and the peptidyl-prolyl cis/trans isomerases putative proteinase maturation protein A (PpmA) and streptococcal lipoprotein rotamase A (SlrA). PpmA and SlrA have a role in the folding or activation of the surface-exposed proteins. The two other candidate lipoproteins, SP_0191 and SP_0899, are so far uncharacterised lipoproteins. With the exception of the uncharacterised lipoproteins SP_0191 and SP_0899, previous studies using *in vivo* mouse models have demonstrated that the selected lipoproteins are involved in virulence (9–11, 14, 28, 32, 41, 42). It can therefore be hypothesised that blocking these antigens, for example through the use of specific antibodies, may lead to the attenuation of virulence and thus confer protection. The lipoprotein-encoding genes were therefore cloned into a pTP1 vector (10) and plasmids were transformed into *E. coli* BL21 for protein expression. The recombinant proteins were purified using affinity chromatography, and their quality and purity was confirmed using the silver staining of an SDS-gel and immunoblotting (Figure 1). All lipoproteins were shown to be stable in solution, and no degradation was observed.

**Table 3:** Sequence homology of selected lipoproteins among different pneumococcal strains based on protein sequences from *S. pneumoniae* TIGR4.

***S. p*. strain**	**AdcAII**	**DacB**	**Etrx1**	**Etrx2**	**MetQ**	**PnrA**	**PpmA**	**PsaA**	**SlrA**	**SP0191**	**SP0899**
TIGR4	100.00	100.00	100.00	100.00	100.00	100.00	100.00	100.00	100.00	100.00	100.00
D39	100.00	99.16	99.47	98.92	99.30	98.29	99.68	99.68	99.25	98.94	99.31
P1031	99.02	98.32	99.47	98.38	99.30	98.86	99.36	99.68	98.88	100.00	99.31
G54	99.02	96.64	100.00	98.92	98.24	98.57	93.15	99.35	99.25	100.00	98.97
Hungary19A	99.67	89.92	100.00	98.92	99.30	98.86	99.68	99.35	98.88	98.41	98.62
70585	99.34	89.08	99.47	100.00	99.65	98.86	100.00	99.68	99.25	100.00	99.31
JJA	100.00	86.97	100.00	100.00	99.65	98.57	99.68	96.76	99.63	100.00	98.89
Taiwan19F	100.00	86.97	100.00	100.00	99.65	98.57	99.68	99.68	99.25	98.94	99.31

### Antigen-specific polyclonal IgGs and the surface abundance of pneumococcal lipoproteins

**Figure 2:**
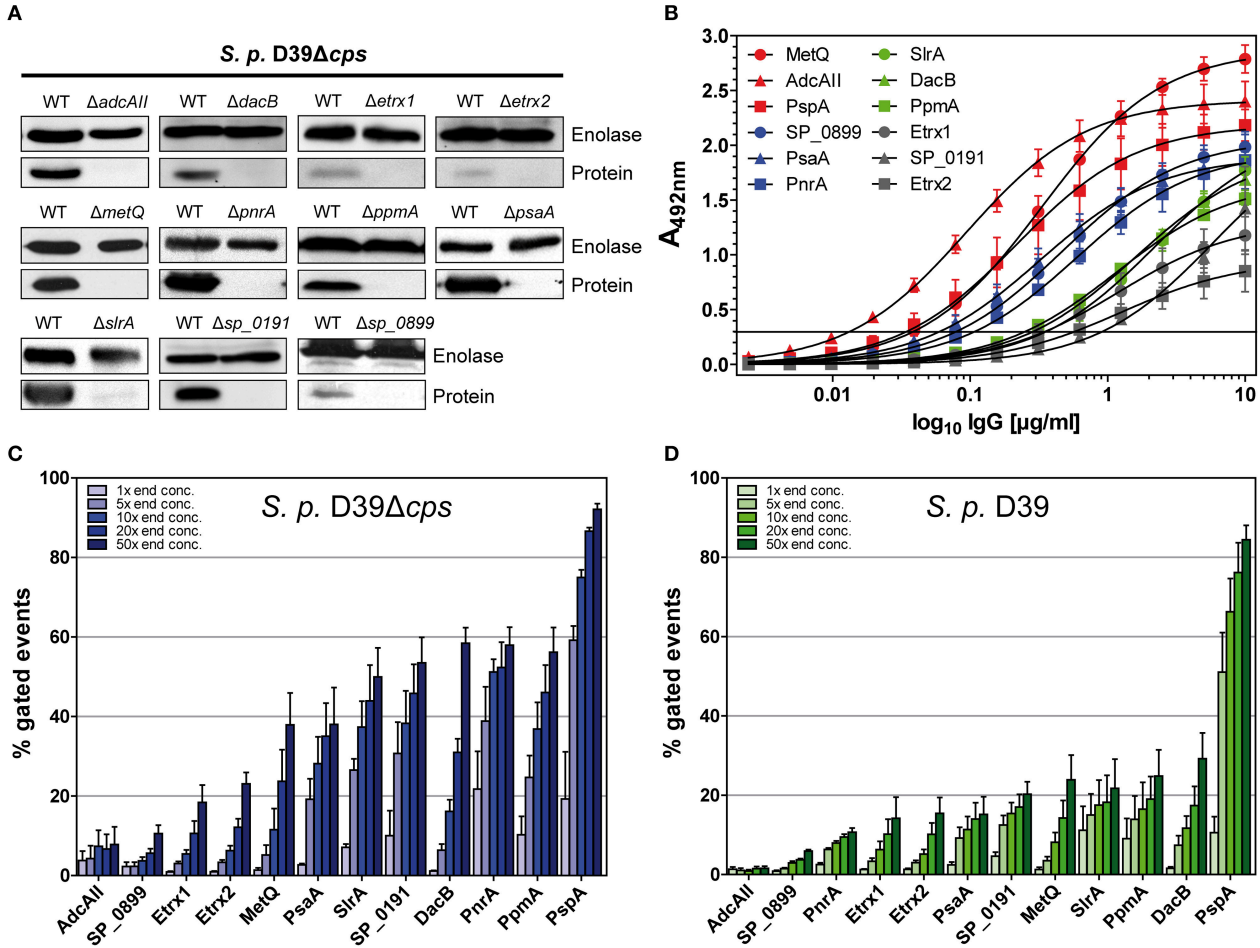
Pneumococcal lipoproteins are highly abundant on the pneumococcal surface. **(A)** In immunoblots, the specificity of antisera derived from intraperitoneal immunisations of CD-1 mice (*n* = 6) with recombinant lipoproteins was assessed. Therefore, the wild-type strain *S. pneumoniae* D39Δ*cps* and the corresponding isogenic lipoprotein deficient mutants (2 × 10^8^ bacteria per lane) were used. Enolase was detected with a rabbit anti-enolase serum and served as a loading control. **(B)** IgG antibody titrations were performed by incubating equimolar amounts of recombinant proteins with serial dilutions of isolated polyclonal IgGs. Detection was carried out using a peroxidase-coupled goat anti-mouse IgG followed by incubation with OPD as a substrate and absorbance was measured at 492 nm. Titrations were performed at least three times and the error bars represent the SEM. **(C,D)** Using the equation for the hyperbolic regression curve ($y\left[Abs\right]=\frac{Bmax\cdot x}{Kd+x}$, Bmax, maximal binding; Kd, concentration for half maximal binding) an initial IgG concentration was calculated in the linear dynamic range. The polyclonal IgGs with equal contents of IgG specific for each lipoprotein were therefore applied to enable the comparison of their surface abundances. In a flow cytometric approach, D39Δ*cps* **(C)** and D39 **(D)** were incubated with the appropriate calculated concentration of IgG and concentrations 5-, 10-, 20-, and 50-fold greater to analyse the surface abundance of the selected lipoproteins. Antibody binding was detected using a goat anti-mouse Alexa Fluor® 488-coupled secondary antibody. The percentage of positive gated events is depicted in the graphs, thereby indicating the proportion of wild-type bacteria positive for the binding of the respective anti-lipoprotein IgGs. The mean values of at least three independent experiments are shown, with error bars corresponding to SEM.

Mice were intraperitoneally immunised with the heterologously expressed lipoproteins to generate antigen-specific antisera. Purified polyclonal IgGs were used for immunoblot analyses, which were performed using the whole-cell lysates of non-encapsulated *S. pneumoniae* D39 and its isogenic lipoprotein-deficient mutants. The immunoblots demonstrated that the anti-lipoprotein IgGs are highly specific; the protein bands were only detected in wild-type pneumococci but not in the corresponding isogenic mutants (Figure 2A). Furthermore, under *in vitro* growth conditions, the protein levels were highly variable, with Etrx1, Etrx2, and SP_0899 showing the lowest levels and PnrA, PsaA, and MetQ the highest levels of expression.

The recognition of pneumococci by the immune system is vital for the host to clear these pathogens. The binding of antigen-specific antibodies depends on the expression, abundance and accessibility of antigens. In order to analyse the surface abundance and accessibility of the selected lipoproteins, we determined the relative antibody titres in mice following immunisation. For this purpose, the recombinant lipoproteins were immobilised in equimolar amounts and incubated with serial dilutions of the polyclonal IgGs, and the initial IgG concentrations were calculated from the resulting hyperbolic titration curves. The highest IgG titres were measured for MetQ, AdcAII, and PspA, while the lowest titres were observed for Etrx1, Etrx2, and SP_0191 (Figure 2B). *S. pneumoniae* D39 and the non-encapsulated mutant D39Δ*cps* were incubated with increasing concentrations of polyclonal IgGs (1×, 5×, 10×, 20×, and 50×) to elucidate the abundance of the 11 selected lipoproteins on the pneumococcal surface using flow cytometry. The initial IgG concentrations calculated in the antibody titration study (Figure 2B), enabled the application of comparable amounts of lipoprotein-specific IgGs in the flow cytometric analysis. We used anti-PspA IgG as a positive control, as it is already known that the choline-binding protein PspA is highly abundant on the surface of these bacteria (43). Overall, the antigen-specific IgGs bound in a dose-dependent manner to the surface of pneumococci (Figure 2C, Table S1). Our data confirm that PspA is probably one of the most abundant pneumococcal surface proteins (Figure 2C, Table S1). When using the highest anti-PspA antibody concentrations, over 80% of the fluorescent pneumococci were detectable. Of our tested lipoproteins, PnrA, PpmA, DacB, SP_0191, and SlrA showed the highest surface abundance, with up to 60% positive fluorescent events, while PsaA and MetQ had lower levels, with approximately 40% of fluorescent pneumococci detectable. The lowest surface abundance was observed for thioredoxins Etrx1 and Etrx2, the putative lipoprotein SP_0899, and the zinc transport system binding protein AdcAII, the latter two of which were almost undetectable. Some of the tested proteins were also accessible for antibody binding when covered by the capsular polysaccharide. DacB, PpmA, SlrA, and MetQ could be detected in the presence of the capsule (Figure 2D, Table S2). However, as expected, the binding capacity was strongly diminished and the positive fluorescent events dropped to 20–30%. Strikingly, the capsule does not block antibody binding to PspA, confirming its exposure and accessibility for antibodies (43). To confirm that IgG binding to *S. pneumoniae* was antigen-specific, mutants deficient for the lipoproteins were incubated with the corresponding polyclonal IgGs. We detected only minor non-specific IgG binding for some of the lipoproteins, indicating that the generated antibodies were overall antigen-specific (Figure S1).

### PnrA, DacB, MetQ, and PsaA Are highly immunogenic

**Figure 3:**
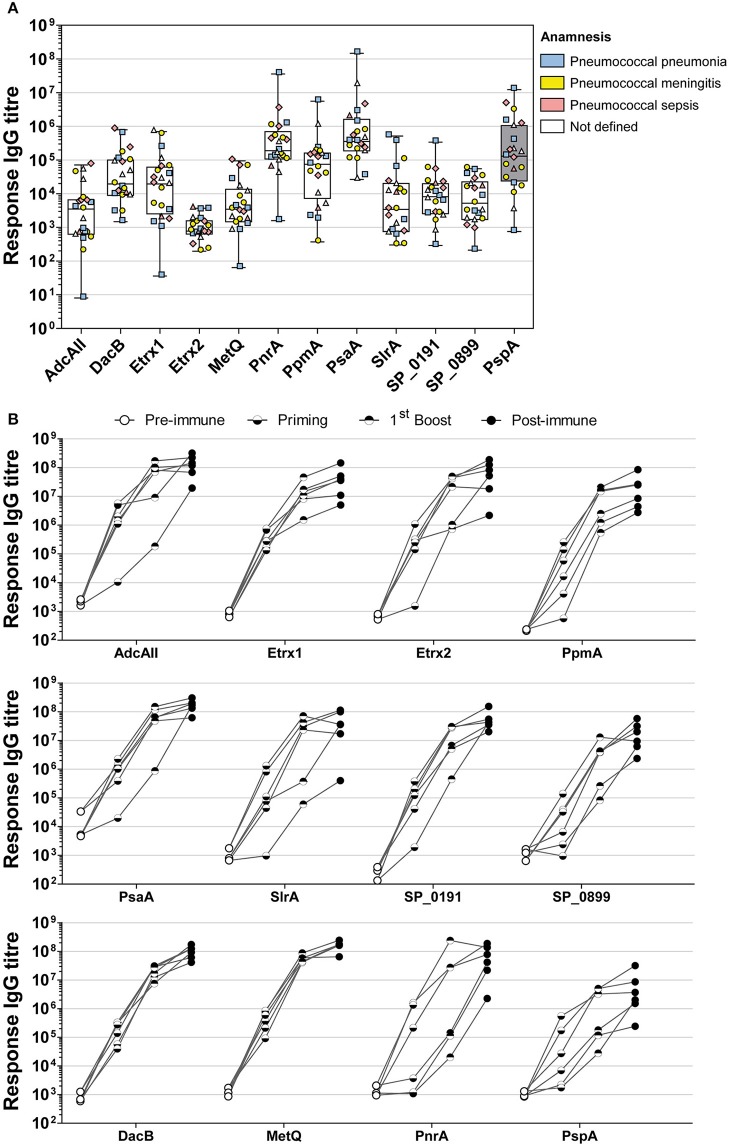
Analysis of convalescent patient sera and mouse sera derived from intraperitoneal immunisations indicate the high immunogenicity of PnrA, DacB, and MetQ. **(A)** A total of 22 antisera from convalescent patients who suffered from pneumococcal infections such as pneumonia (*n* = 6), meningitis (*n* = 7), sepsis (*n* = 4), and unknown clinical outcomes (*n* = 5) caused by different pneumococcal serotypes were analysed to compare their levels of anti-lipoprotein antibodies. Each symbol represents a single antiserum, while the different colours indicate the clinical outcome of every patient. **(B)** The immunogenicity of the lipoproteins was further demonstrated by analysing the antibody kinetics of intraperitoneally immunised CD-1 mice (*n* = 6). The mice received three vaccinations with 20 μg antigen and Alum as the adjuvant, with a 2-week interval between treatments. Before each treatment and 2 weeks after the final immunisation, antisera were collected to enable the determination of the antibody kinetics. Each individual mouse is depicted in the graphs. All serum samples were serially diluted and measured using the FLEXMAP 3D® system. Response values reflect the levels of antigen-specific IgG for the 11 tested lipoproteins and the positive control PspA.

The immunogenicity of the selected lipoproteins was investigated using multiplex immunoassay technology. Two different types of sera were analysed: (i) convalescent patient sera and (ii) antisera from mice intraperitoneally immunised with pneumococcal antigens using Alum as the adjuvant (Figure 3). Recombinant proteins covalently coupled to fluorescent MagPlex® beads were incubated with serial dilutions of human or mouse sera. Measurements of convalescent patient sera revealed the highest IgG levels for PsaA and PnrA, which were both comparable to those of the positive control, PspA (Figure 3A). DacB, PpmA, and Etrx1 IgG levels were also high, although they were an order of magnitude lower than those for PsaA. The titres of antibodies for Etrx2, AdcAII, MetQ, SP_0191, SP_0899, and SlrA were comparatively low in randomly selected convalescent sera. The final antibody titres of mouse sera obtained 2 weeks after the third and final immunisation ranged from 2.9 × 10^6^ AU (α-PspA) to 1.8 × 10^8^ AU (α-PsaA), and were at least three orders of magnitude higher than the titres of the pre-immune sera. The highest antibody titres were measured for PsaA, MetQ, AdcAII, and DacB. The second boost did not substantially increase the antibody titres, which were on average only 2.5-fold higher than the levels measured after the first booster immunisation. Notably, the changes in antibody titres for MetQ and DacB were highly similar when compared between individual mice. However, the antibody titres for the majority of the other lipoproteins varied substantially. In conclusion, lipoproteins such as DacB, MetQ, and PnrA represent promising candidates for the development of a robustly effective and reliable vaccination.

### Intranasal vaccination with DacB, MetQ, or PnrA reduces pneumococcal load in the nasal cavity

**Figure 4:**
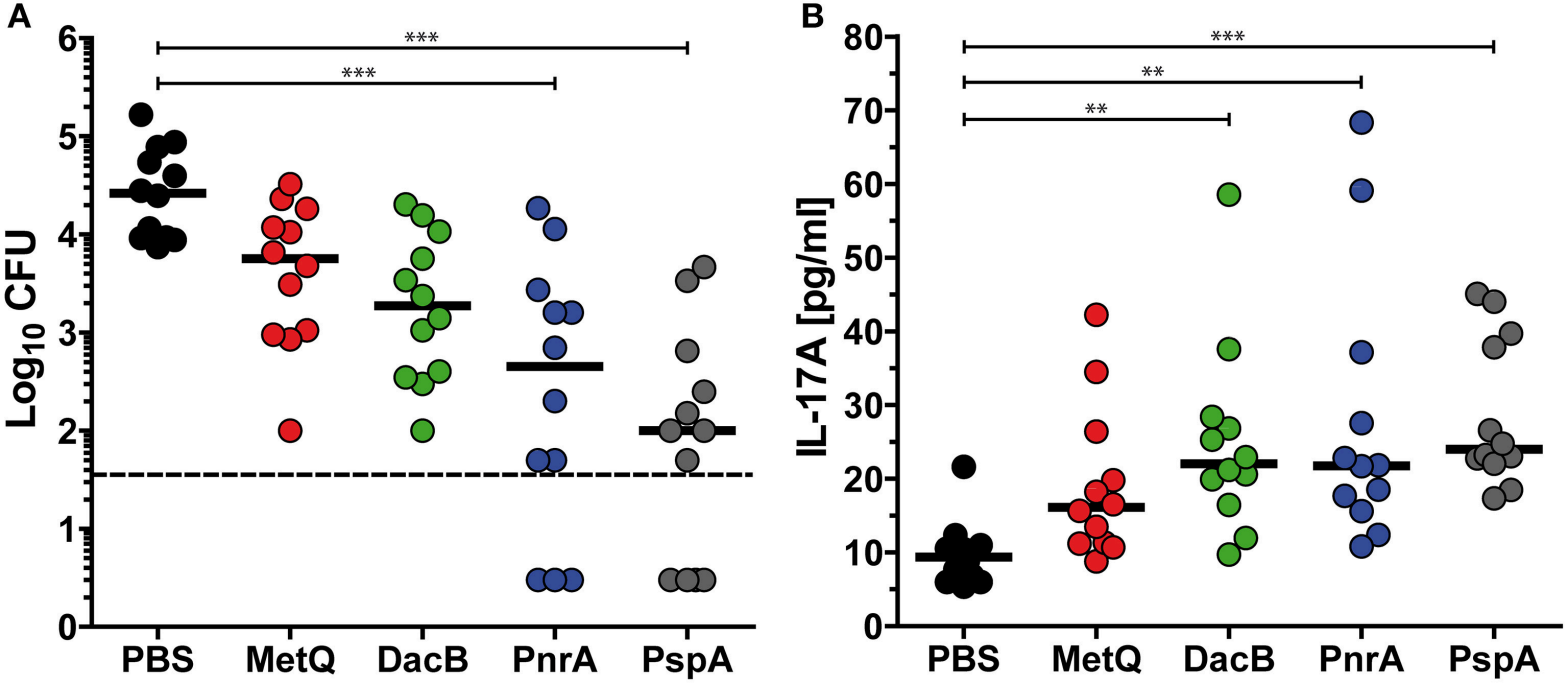
Intranasal vaccinations with the lipoproteins MetQ, DacB or PnrA reduce pneumococcal colonisation and increase local IL-17A levels. Bacterial recovery of *S. pneumoniae* D39 from nasal tissue **(A)** and nasopharyngeal IL-17A levels **(B)** 3 days after the intranasal challenge of C57BL/6 mice (*n* = 12) with 3.4 × 10^6^ CFU. Each mouse received three intranasal immunisations with 5 μg of one of the four recombinant proteins, MetQ, DacB, PnrA, or PspA, in combination with 4 μg CTB in 2-week intervals. The data were statistically analysed using a Kruskal Wallis test accompanied by Dunn's multiple comparison post-test, with all conditions compared to control mice that received an intranasal treatment with PBS and CTB. Symbols indicate individual mice, bars represent the group median, and the dotted line indicates the lower limit of detection. **p < 0.01; ***p < 0.001.

Based on the previous screenings, three lipoproteins DacB, PnrA, and MetQ were selected to assess whether their use in an intranasal vaccination would confer protection against pneumococcal colonisation. Mice were intranasally immunised with the protein candidates in combination with CTB as an adjuvant, and received two booster immunisations. PspA was included as a positive control, while PBS mock-treated mice were used as a negative control. Three weeks after the final vaccination, the mice were intranasally infected with a non-lethal dose of 3.4 × 10^6^ *S. pneumoniae* D39. Three days post-infection, mice were euthanised and live pneumococci were recovered from their nasal tissues. Consistent with previous studies, an intranasal vaccination with PspA induced the strongest reduction (263-fold) of bacterial load in the nasal cavity when comparing the bacterial load to mock-treated mice (Figure 4A). PnrA also showed strong efficacy, causing a 58-fold reduction in the number of *S. pneumoniae* in the nasopharynx. Of the mice, which received the PnrA immunisation, 50% had almost completely cleared the pneumococci within 3 days post-infection. Immunisation with DacB and MetQ showed a trend in reducing the bacterial load. However, reduction was only 14- or 4-fold, respectively, and not statistically significant.

### Protective immunity correlates with increased intranasal IL-17A levels

Immunity to *S. pneumoniae* infection was shown to be dependent on the induction of IL-17A-secreting CD4^+^ T cells, leading to the recruitment of neutrophils to enable the clearance of the pneumococci (44–46). To determine the role of IL-17A in our protection studies, we quantified the levels of this cytokine in the nasal tissues of mice immunised with DacB, MetQ, or PnrA 3 days after infection. Significantly increased IL-17A levels were identified in the DacB- and PnrA-immunised mice in comparison with the negative control, both reaching IL17A levels comparable to mice immunised with PspA (Figure 4B). Importantly, production of nasopharyngeal IL-17A significantly correlated with the level of protective immunity induced by the pneumococcal lipoproteins (ρ = −0.3916; *p* = 0.002), which was indicated by a Spearman correlation test (Figure S2). These results confirm the important role of IL-17A in protecting against pneumococcal colonisation.

### Intranasal immunisation only partially induces local and systemic antigen-specific antibodies

**Figure 5:**
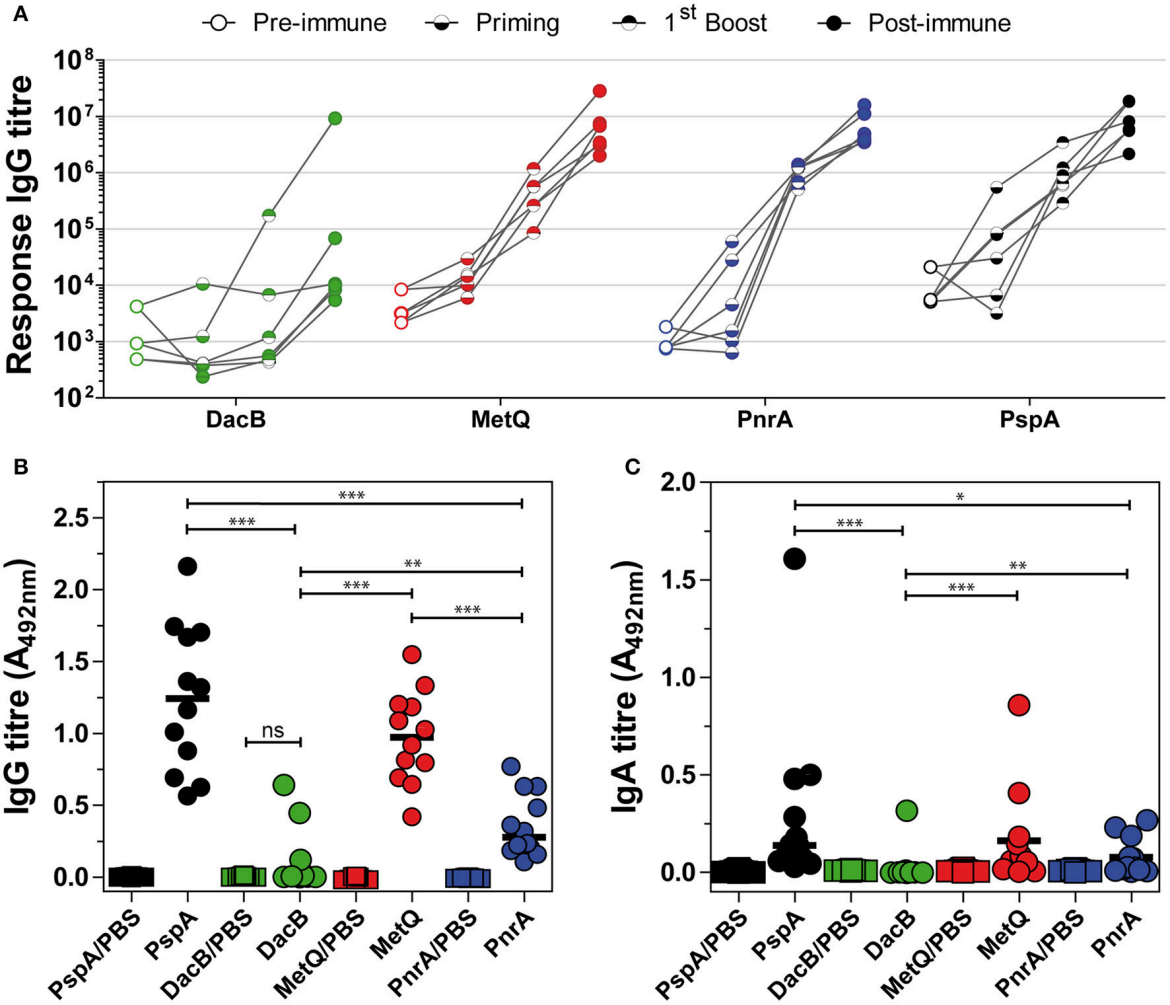
Intranasal immunisation with lipoproteins induces lower local and systemic humoral immune responses. **(A)** Six C57BL/6 mice used for the *in vivo* colonisation model were randomly selected for the analysis of their antibody kinetics following an intranasal immunisation with the lipoproteins MetQ, DacB, and PnrA. The mice received three doses with 5 μg antigen and 4 μg CTB as the adjuvant in 2-week intervals. Before each treatment and 2 weeks after the third immunisation, antisera were collected to determine the antibody kinetics. The data from each individual mouse are depicted for every protein. Antisera were serially diluted and measured using the FLEXMAP 3D® system. The response values reflect the levels of antigen-specific IgG. **(B,C)** Three weeks after the final immunisation, the mice were challenged with *S. pneumoniae* D39 (3.4 × 10^6^ CFU) and 3 days after infection their nasal tissues were harvested, homogenised and analysed for local antigen-specific IgG **(B)** and IgA **(C)** using ELISA. The IgG and IgA levels were determined using a 1:10 or 1:2 dilution of the nasal homogenate, respectively. The data were statistically analysed using a Mann-Whitney *U*-test. Symbols represent individual mice (*n* = 12) and the bars represent the group median. *p < 0.05; **p < 0.01; ***p < 0.001.

Humoral immune responses following intranasal immunisation with recombinant lipoproteins were investigated by analysis of antibody kinetics in post-immune antisera and the local antibody titres in nasal tissues harvested 3 days after infection with pneumococci. As shown in Figure 5A, intranasal immunisation with recombinant MetQ, PnrA, and PspA induced strong systemic antigen-specific antibody responses in all tested mice. However, a substantial systemic IgG response for recombinant DacB was only detected in one of the six mice. Three days after infection, we analysed the local humoral immune responses in the nasal tissues of all mice used in the model of colonisation (*n* = 12) to elucidate whether lipoprotein-specific immunoglobulins are present in the nasal cavity, which might contribute to protection (Figures 5B,C). MetQ and PspA were found to be potent immunogens when administered intranasally, as demonstrated by the resulting high local titres of IgG, while the local IgG response for PnrA was significantly lower (Figure 5B). The local IgG response for DacB was substantially lower than MetQ and PspA, which was consistent with the antibody kinetics. Intranasal immunisation with PspA or MetQ induced considerable levels of local antigen-specific IgA, whereas nasal IgA for PnrA and DacB were almost too low to be detected (Figure 5C). Taken together, these data suggest that local and serum antigen-specific antibody responses might not perfectly correlate with protection *in vivo*, as was especially shown for PnrA and DacB.

### Immunisations with DacB, MetQ, or PnrA predominantly induce IgG1 responses

**Figure 6:**
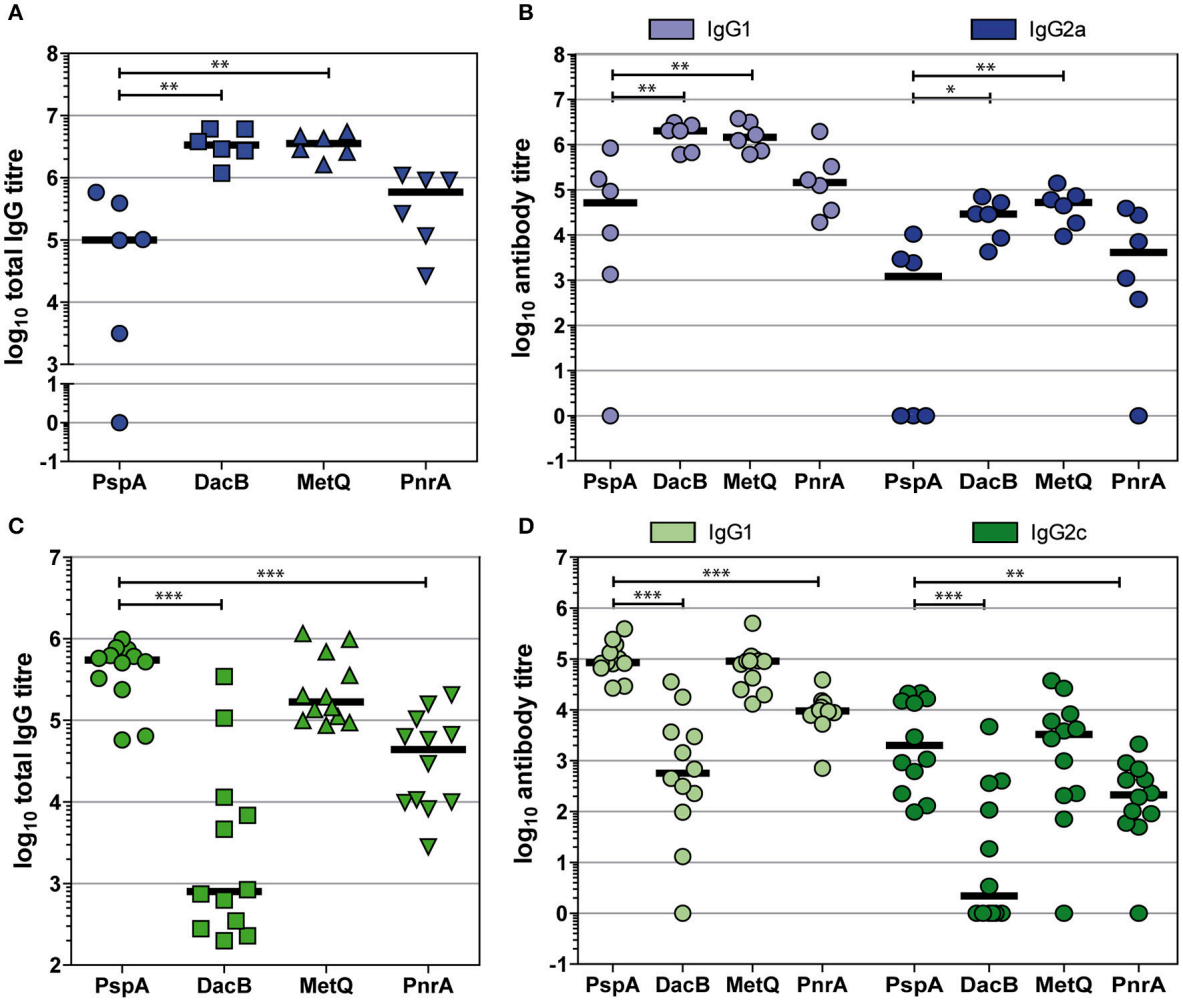
Intraperitoneal and intranasal immunisations with DacB, MetQ, or PnrA predominantly induce IgG1 responses. Antigen-specific total IgG, IgG1, and IgG2 titres were monitored using an ELISA in either post-immune sera following an intraperitoneal immunisation with Alum as the adjuvant **(A,B)** or in post-challenge sera obtained after intranasal immunisation with CTB as the adjuvant followed by an intranasal challenge with *S. pneumoniae* D39 **(C,D)**. Antibody titres of each serum specimen are denoted as the log_10_ of the reciprocal dilution of the serum giving twice the average absorbance of the sera derived from the PBS-treated group. The data were statistically analysed using a Mann-Whitney *U*-test. Symbols represent individual mice (*n* = 6 for Alum group, *n* = 12 for CTB group) and bars represent the group median. *p < 0.05; **p < 0.01; ***p < 0.001.

To shed light on the type of immune response induced by the intraperitoneal or intranasal vaccinations with our candidate proteins in combination with Alum or CTB as the adjuvant, respectively, we determined the IgG1 and IgG2a/IgG2c levels in post-immune sera (Figure 6). Overall, intraperitoneal immunisation with the lipoproteins DacB, MetQ and PnrA elicited higher total IgG responses than intranasal immunisation (Figures 6A,C). Immunising mice either intranasally or intraperitoneally with the four pneumococcal antigens predominantly led to high IgG1 and lower but still substantial levels of IgG2, suggesting a Th2-biased response (Figures 6B,D). The intranasal immunisation with DacB resulted in a remarkably high IgG1/IgG2 ratio and a very weak humoral immune response overall (Figure 6B). In summary, intranasal immunisation with the lipoproteins DacB, MetQ, and PnrA reduced pneumococcal colonisation, and the level of protection correlated with IL-17A levels. However, immunisation only partially induced local and systemic antigen-specific antibody responses.

## Discussion

Pneumococcal colonisation of the upper respiratory tract is a prerequisite for invasive disease (47). Higher colonisation rates facilitate the transmission of this opportunistic pathogen from host to host, enabling pneumococci to spread within a population, as was shown in influenza A co-infection mouse models (48). Protein-based vaccines should therefore include one or more antigens that reduce nasopharyngeal colonisation to prevent infectious diseases and the shedding of pneumococci. In order to elicit serotype-independent protection, conserved pneumococcal surface proteins are of special interest. Previous studies have already shown that PsaA, a highly conserved manganese-binding lipoprotein, provides cross-protection in a mouse model of colonisation following intranasal immunisation (23). Here, we selected 11 pneumococcal lipoproteins with high sequence homology (>85%) and investigated their potential for use as subunits of such a vaccine.

The expression and surface accessibility of potential vaccine candidates is important for the host immune system to recognise and counteract the dissemination of pneumococci to normally sterile body sites. Under *in vitro* growth conditions in a rich medium, the highest surface abundance of lipoproteins was observed for PpmA, PnrA, and DacB, while AdcAII and the thioredoxins Etrx1 and Etrx2 were detected at a substantially lower abundance. The highest surface abundance was shown for the choline-binding protein PspA, a major virulence factor of pneumococci known to be highly immunogenic and abundant on the surface (43, 49). The pneumococcal capsular polysaccharide (CPS) is known to mask surface-exposed antigens, thereby blocking opsonisation by inhibiting antibody binding and consequently limiting pathogen uptake by professional phagocytes. Indeed, we also indicate that the detection of surface-localised lipoproteins was decreased when using the encapsulated strain D39, while the CPS only marginally diminished antibody binding to PspA, consistent with the findings of previous studies (43). The presence of the CPS could not completely inhibit antibody binding to the surface-exposed lipoproteins, however, as indicated by the dose-dependent increase of fluorescence intensities in the flow cytometric analysis. In previous studies, the iron uptake ABC transporter lipoproteins PiaA and PiuA and the nucleoside-binding lipoprotein PnrA were shown to be surface accessible. Indeed, antigen-specific antibodies bound to encapsulated pneumococci, but cross-reactivity to heterologous strains was only demonstrated for the PnrA-specific antibodies, suggesting that PnrA is conserved across pneumococcal serotypes (11, 50). It was previously reported that anti-PsaA and anti-PpmA failed to detect PsaA and PpmA on the surface of different pneumococcal strains (49), in contrast to our findings. The differences in the accessibilities of the analysed lipoproteins could be due to the variable capsular structures and expression levels, their localisation in the cell wall, and especially the different levels of expression for each lipoprotein gene. The latter point must be seriously evaluated when searching for a new protein-based vaccine, because several studies have shown that pneumococcal gene expression is highly dependent on the strain and the host compartment in which the pneumococci reside (51–55).

To evaluate the humoral immune responses induced by the selected pneumococcal lipoproteins, a multiplex bead-based immunoassay was performed. Our analysis of convalescent patient sera revealed high antibody titres for the lipoproteins PsaA, PnrA, PpmA, and DacB. Therefore, we concluded that these lipoproteins are immunogenic during natural infections. PspA elicited an exceptionally high humoral immune response, as also reported previously (36, 56). We further investigated the immunogenicity of the selected lipoproteins in an immunisation study using intraperitoneally vaccinated mice. High endpoint antibody titres were measured for PsaA, DacB, PnrA, MetQ, and AdcAII. Immunisation with DacB and MetQ was particularly effective, rapidly increasing the antibody titres in all mice. In most cases the first booster immunisation was sufficient to elicit high antibody responses, suggesting that a two-dose immunisation strategy may be sufficient for accomplishing an optimal humoral immune response. Taken together, these data show that pneumococcal lipoproteins are generally highly immunogenic. Lipoproteins are able to elicit humoral immune responses during natural pneumococcal colonisation or infections, as shown for PsaA, PnrA, and DacB (20–22, 56, 57), and may also be used in immunisation to induce a substantial immune response. These findings are strongly supported by several other studies using different lipoproteins as vaccine antigens, including PsaA, PnrA, PiuA, and PiaA (11, 23, 58).

As mentioned above, colonisation is the first step towards the establishment of pneumococcal infections Hence, protection against colonisation is a crucial aspect of pneumococcal vaccine development. Based on the surface abundances, accessibility and immunogenicity of the lipoproteins, we selected DacB, MetQ, and PnrA for the assessment of their protective potential in a mouse model of colonisation. Subcutaneous immunisation with PnrA was previously shown to induce protective immunity against an intraperitoneal challenge with heterologous *S. pneumoniae* strains (11). In the present work, we further confirmed the potential of PnrA as a component of protein-based subunit vaccines. Its use in the intranasal immunisation of mice significantly decreased the bacterial loads in the nasal cavity. Immunization with DacB and MetQ only tended to reduce bacterial load in the nasal cavity to an insignificant extent. Basavanna et al. indicated that systemic vaccination with MetQ does not extend the survival or result in differences in the progression of fatal infections. Based on a transcriptome analysis, they reasoned that this lack of protection might be due to the low expression of *metQ* in infection-related niches (12). This might be the critical factor causing the comparatively low protective effect of MetQ immunisation against pneumococcal colonisation in our study.

The pro-inflammatory cytokine IL-17, among others secreted by Th17 cells, is essential for recruitment and activation of macrophages and neutrophils to the nasopharynx, a process critical for clearing pneumococci from the host (59). Th17-mediated immunity is essential for protection against pneumococcal colonisation, as CD4^+^ T cell-derived IL-17, but not IFNγ or IL-4, is required for the clearance of colonisation (46). Furthermore, intranasal immunisation with pneumococcal whole-cell antigens or a subunit-protein vaccine was found to provide IL-17-mediated, but antibody-independent, protection (45). Here, we monitored that the nasal tissues of mice vaccinated with DacB, PnrA, and PspA showed significantly increased IL-17A levels 3 days after infection with pneumococci that correlated with protection. These elevated local IL-17A concentrations probably result from recall responses of immunization-induced memory towards these antigens. Accordingly, the immunisation of mice with MetQ, which caused the lowest reduction in bacterial load, provoked only a slight increase in IL-17A levels. Consistent with our results, strong correlations between high IL-17A levels and protection against colonisation have been reported in previous studies, where increased *ex vivo* IL-17A was predictive of *in vivo* nasal IL-17A levels following vaccination and, furthermore, was an indicator of protective efficacy (60, 61).

CTB is a potent adjuvant with various immunomodulatory functions, which are mainly attributed to its ability to bind to monosialotetrahexosylganglioside (GM1). GM1 is broadly distributed in a variety of cell types, including the epithelial cells of the gut and antigen-presenting cells, macrophages, dendritic cells, and B cells. Therefore, CTB can enhance the immune responses to bystander antigens, a phenomenon indicated by the production of effective antigen-specific antibodies at the mucosal surfaces (62–65). Immunity to pathogens at mucosal surfaces is especially driven by antigen-specific secretory IgA (sIgA), which acts as an inhibitor of adherence and inflammation and is able to neutralise viruses, toxins and enzymes (66–70). Here, we showed that intranasal immunisation with PspA plus CTB had the strongest effect on the reduction of pneumococcal load in the nasal cavity. We could only detect considerable local IgA levels in a few mice immunised with PspA and CTB, although they were still significantly higher than those found for DacB and PnrA. In contrast, substantial amounts of nasal IgG were detected for PspA and MetQ, though significantly less DacB- and PnrA-specific local IgG was identified. After either intranasal immunisation with CTB followed by a challenge with *S. pneumoniae* D39 or intraperitoneal immunisation with Alum as the adjuvant, the systemic humoral immune responses varied depending on the antigen and route of immunisation. Overall, they were characterised by a predominance for IgG1 and substantial IgG2a/IgG2c production, suggesting a primary Th2 response, which is consistent with previous studies and was attributed to the use of Alum as adjuvant (63, 71, 72). While intraperitoneal immunisation with DacB and Alum as an adjuvant led to a strong systemic antibody response, the intranasal administration of DacB in combination with CTB induced only marginal levels of IgG production. The opposite was observed for PspA, where intranasal immunisation provoked higher antibody titres compared with the intraperitoneal administration. It is unclear why PspA is a potent immunogen when administered in combination with CTB via the nasal route but less immunogenic in a systemic vaccination using Alum as an adjuvant; however, our results are in accordance with a previous study using a different mouse strain and a slightly different immunisation protocol (73). It has been reported that the efficient induction of an immune response depends on the adjuvant, the route of immunisation and the immunogenicity of the antigen itself (74, 75). Both Alum and CTB represent potent adjuvants, as the antibody titres for at least two proteins in our vaccinations were highly elevated. This further indicates that, in principle, our tested lipoproteins are immunogenic, a fact supported by the analysis of convalescent patient sera. It therefore seems likely that the route of immunisation has a profound role on the magnitude of the immune response. In a vaccination study where rats were immunised with three structurally different types of pneumococcal polysaccharide (PPS-3, PPS-4, and PPS-14) using four immunisation routes, remarkable differences were observed in both the magnitude of the immune response and the distribution of the isotypes (76). The authors concluded that, besides the route of immunisation, the structural features of the pneumococcal polysaccharides have a pivotal influence on the elicited immune response. Likewise, structural differences of the proteins could be one of the reasons for the varying immune responses *in vivo*. Although intranasal vaccination with DacB could not induce high levels of antigen-specific antibody production, it had a drastic effect on the reduction of pneumococcal colonisation accompanied by elevated local levels of IL-17. This suggests that protection is rather characterised by a cellular immune response mediated by local antigen-specific CD4^+^ memory T cells than by a humoral immune response. Accordingly, in a previous study it was shown that protection against pneumococcal colonisation by intranasal immunisation with three pneumococcal proteins (PspC, PsaA, and PdT) was dependent on CD4^+^ T cells but independent of antibodies (45). It therefore remains unclear whether local or systemic antibody responses, especially towards DacB, result in the protective effect in the mouse model of colonisation following intranasal immunisation.

In conclusion, we showed that vaccination of mice with a monovalent protein-based vaccine containing the lipoprotein PnrA impairs nasopharyngeal colonisation by pneumococci after intranasal challenge with *S. pneumoniae* D39. There was a possible protective effect for DacB and MetQ as vaccine candidates, although it was less pronounced and not significant. The lipoproteins evaluated here are highly conserved among pneumococcal serotypes, abundant on the pneumococcal surface, and immunogenic. These properties mean they are promising protein antigens for a next-generation subunit vaccine for the reduction of pneumococcal colonisation, which could be accompanied by a decline in the transmission of pneumococcal infections. Future studies are required to elucidate the mechanisms of protective immunity induced by these lipoproteins and to identify the optimal route of immunisation and appropriate adjuvant.

## Author contributions

FV, MdJ, and SH conceived and designed the experiments. FV, TK, TM, FvO, MA, MS, and SvS performed the experiments. FV, TM, SM, FS, and SH analysed the data. FV, MdJ, and SH wrote the manuscript.

### Conflict of interest statement

The authors declare that the research was conducted in the absence of any commercial or financial relationships that could be construed as a potential conflict of interest.
