# Supplementary material for: Intranasal Vaccination With Lipoproteins Confers Protection Against Pneumococcal Colonisation
Source: Front Immunol. 2018 Oct 18;9:2405. doi: 10.3389/fimmu.2018.02405 (PMC6202950; doi:10.3389/fimmu.2018.02405)
Supplement: Supplementary file 1 [file Data_Sheet_1.pdf]

Supplementary Material

Intranasal vaccination with lipoproteins confers protection against pneumococcal colonization

Franziska Voß[1], Thomas P. Kohler[1], Tanja Meyer[2], Mohammed R. Abdullah[1], Fred J. van Opzeeland[3], Malek Saleh[1], Stephan Michalik[2], Saskia van Selm[3], Frank Schmidt[2,4], Marien I. de Jonge[3] and Sven Hammerschmidt[1*]

[1] Department of Molecular Genetics and Infection Biology, Center for Functional Genomics of Microbes, Interfaculty Institute of Genetics and Functional Genomics, University of Greifswald, Greifswald, Germany, [2] Department of Functional Genomics, Center for Functional Genomics of Microbes, Interfaculty Institute of Genetics and Functional Genomics, University Medicine Greifswald, Greifswald, Germany, [3] Section Pediatric Infectious Diseases, Laboratory of Medical Immunology, Radboud Center for Infectious Diseases, Radboud Institute for Molecular Life Sciences, Radboud University Medical Center, Nijmegen, The Netherlands, [4] ZIK-FunGene, Department of Functional Genomics, Interfaculty Institute for Genetics and Functional Genomics, University Medicine Greifswald, Greifswald, Germany

*** Correspondence:** Prof. Dr. Sven Hammerschmidt: sven.hammerschmit@uni-greifswald.de

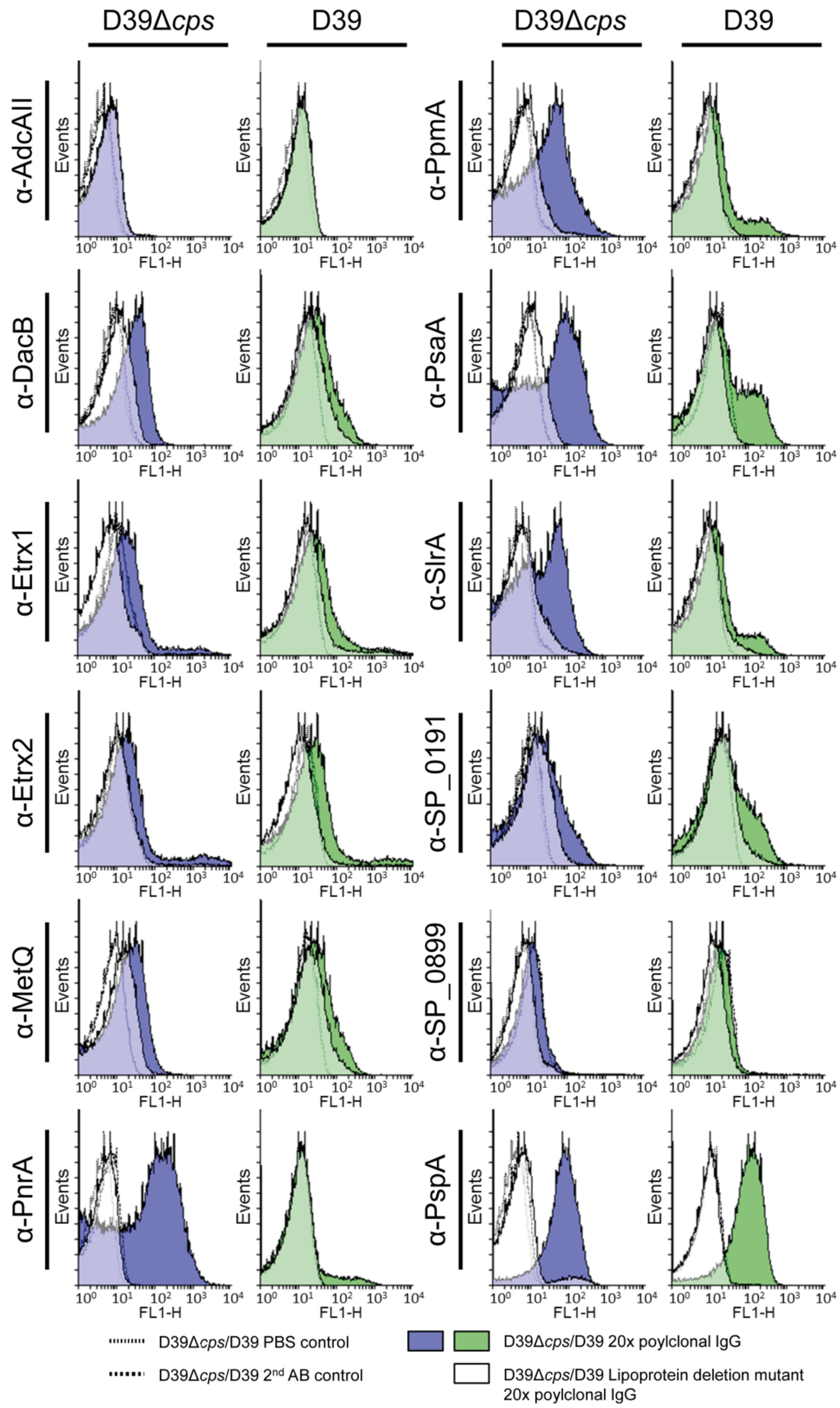

Figure S1. Specificity of anti-lipoprotein specific polyclonal IgGs using flow cytometric analysis. *S. pneumoniae* D39 with or without capsule and the corresponding isogenic lipoprotein deletion mutants were incubated with the 20-fold concentration of the appropriate anti-lipoprotein IgG. Pneumococci treated with PBS or the secondary antibody were used as a negative control. Antibody binding was detected using a goat anti-mouse Alexa Fluor® 488-coupled secondary antibody and is indicated by increased fluorescence intensities (FL1-H) in the histograms.

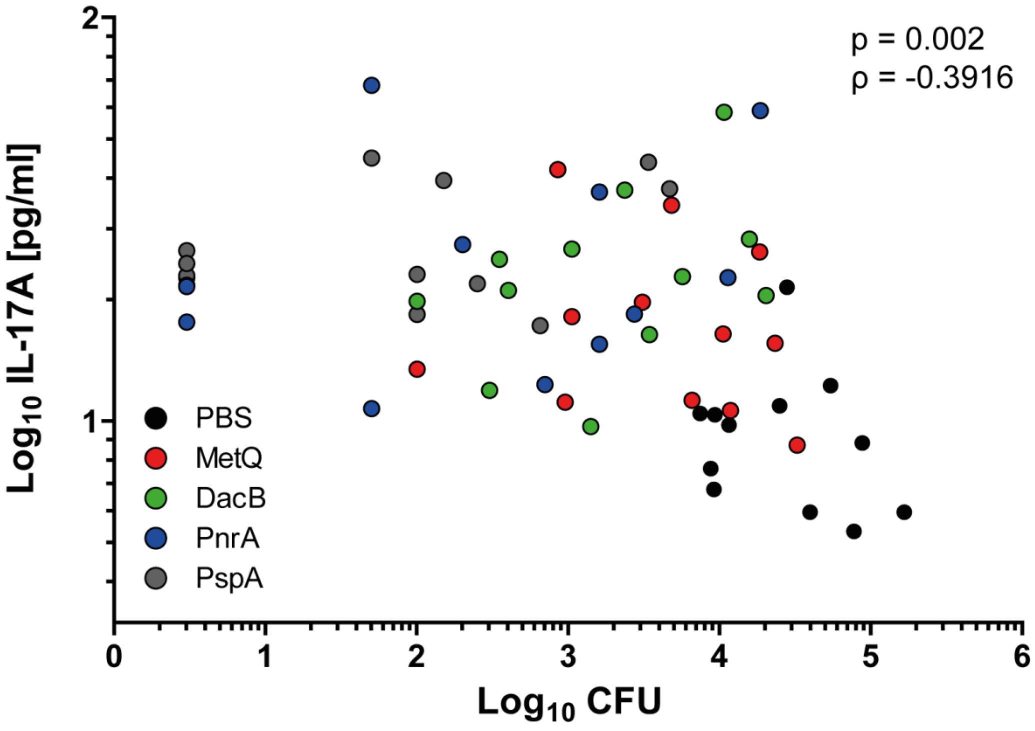

Figure S2: Protective immunity correlates with intranasal IL-17A levels. Paired analysis of CFU counts and nasopharyngeal IL-17A three days post-infection in mice immunized three times with 5 µg recombinant proteins MetQ, DacB, PnrA or PspA in combination with CTB as adjuvant. Analysis was performed with log-transformed data. Symbols represent individual mice (n = 12 per group). Spearman's coefficient (ρ) and p-value are indicated.

Table S1. Surface abundance and accessibility of pneumococcal lipoproteins in *S. pneumoniae* D39Δ*cps*.

Polyclonal IgG	% *S. p.* D39Δ*cps* positive for IgG*									
	1x end concentration of polyclonal IgG		5x end concentration of polyclonal IgG		10x end concentration of polyclonal IgG		20x end concentration of polyclonal IgG		50x end concentration of polyclonal IgG	
	Mean	SEM	Mean	SEM	Mean	SEM	Mean	SEM	Mean	SEM
**AdcAII**	3.76	2.35	4.25	3.25	7.35	4.04	6.65	3.70	7.81	4.42
**DacB**	2.28	1.06	2.31	1.00	3.70	0.94	5.64	1.12	10.53	2.12
**Etrx1**	0.88	0.18	3.00	0.56	5.41	1.03	10.62	3.09	18.36	4.37
**Etrx2**	0.93	0.23	3.29	0.64	6.28	1.23	12.11	2.15	23.05	2.88
**MetQ**	1.36	0.58	5.15	2.47	11.52	5.35	23.70	7.94	37.93	7.99
**PnrA**	2.66	0.37	19.19	5.13	28.13	6.77	35.03	8.32	38.02	9.26
**PpmA**	7.07	0.87	26.51	2.82	37.31	6.52	43.91	9.05	49.93	7.31
**PsaA**	10.01	6.30	30.73	7.84	38.26	8.18	45.83	7.30	53.48	6.41
**SlrA**	1.16	0.13	6.36	1.56	16.10	2.98	30.98	3.40	58.42	3.92
**SP0191**	21.75	9.46	38.82	8.67	51.19	3.20	52.31	6.40	57.91	4.58
**SP0899**	10.24	4.65	24.69	5.49	36.86	6.69	46.02	6.94	56.15	6.22
**PspA**	19.25	11.86	59.18	3.56	74.97	1.94	86.62	0.90	92.10	1.43

* Based on antibody titrations, initial polyclonal IgG concentrations were calculated in the linear dynamic range using the equation $y[Abs] = \frac{Bmax \cdot x}{Kd + x}$ (Bmax: maximal binding, Kd: concentration for half maximal binding). In a flow cytometric approach, D39Δ*cps* was incubated with the calculated 1x IgG concentration and its 5-, 10-, 20- and 50-fold amount in order to analyze the surface abundance of selected lipoproteins. Antibody binding was detected using a goat anti-mouse Alexa Fluor® 488-coupled secondary antibody. The proportion of wild-type bacteria positive for binding of the respective anti-lipoprotein IgG is indicated by the mean values of at least three independent experiments with the corresponding SEM.

Table S2. Surface abundance and accessibility of pneumococcal lipoproteins in *S. pneumoniae* D39.

Polyclonal IgG	% *S. p.* D39 positive for IgG									
	1x end concentration of polyclonal IgG		5x end concentration of polyclonal IgG		10x end concentration of polyclonal IgG		20x end concentration of polyclonal IgG		50x end concentration of polyclonal IgG	
	Mean	SEM	Mean	SEM	Mean	SEM	Mean	SEM	Mean	SEM
**AdcAII**	1.35	0.54	1.03	0.58	0.90	0.32	1.50	0.53	1.56	0.54
**DacB**	0.87	0.20	1.50	0.19	2.89	0.48	3.74	0.27	5.93	0.38
**Etrx1**	2.54	0.40	6.32	0.37	7.96	0.54	9.46	0.72	10.66	1.07
**Etrx2**	1.27	0.17	3.34	0.80	6.31	2.05	10.18	3.77	14.18	5.31
**MetQ**	1.28	0.31	3.02	0.56	5.18	1.14	10.14	2.86	15.45	3.97
**PnrA**	2.45	0.72	9.18	2.71	11.36	3.28	13.98	4.13	15.14	4.44
**PpmA**	4.64	1.00	12.47	2.46	15.39	2.77	17.03	3.21	20.28	3.12
**PsaA**	1.25	0.57	3.50	1.00	8.13	2.47	14.27	4.43	23.89	6.24
**SlrA**	11.15	6.05	15.00	5.33	17.47	6.32	18.22	6.82	21.70	7.39
**SP0191**	9.04	5.09	13.93	5.87	16.50	6.73	18.99	5.73	24.82	6.65
**SP0899**	1.56	0.40	7.40	2.40	11.70	3.04	17.36	4.83	29.19	6.51
**PspA**	10.57	4.04	51.04	9.94	66.23	8.41	76.16	7.53	84.42	3.62

* Based on antibody titrations, initial polyclonal IgG concentrations were calculated in the linear dynamic range using the equation $y[Abs] = \frac{Bmax \cdot x}{Kd + x}$ (Bmax: maximal binding, Kd: concentration for half maximal binding). In a flow cytometric approach, D39 was incubated with the calculated 1x IgG concentration and its 5-, 10-, 20- and 50-fold amount in order to analyze the surface abundance of selected lipoproteins. Antibody binding was detected using a goat anti-mouse Alexa Fluor® 488-coupled secondary antibody. The proportion of wild-type bacteria positive for binding of the respective anti-lipoprotein IgG is indicated by the mean values of at least three independent experiments with the corresponding SEM.
